# Occurrence and seasonal variation of aflatoxin M_1_ in raw cow milk collected from different regions of Algeria

**DOI:** 10.14202/vetworld.2020.433-439

**Published:** 2020-03-09

**Authors:** Sarah Mohammedi-Ameur, Mohammedi Dahmane, Carlo Brera, Moustafa Kardjadj, Meriem Hind Ben-Mahdi

**Affiliations:** 1 Laboratory of Animal Health and Productions, Higher National Veterinary School, Algiers, Algeria; 2 High School of Food Sciences and Food Industries (ESSAIA), Algiers, Algeria; 3 Department of Food Safety, Nutrition and Veterinary Public Health, Food Chemistry Unit, Italian National Institute of Health (ISS), Viale Regina Elena, 299, Rome, Italy

**Keywords:** aflatoxin M_1_, Algeria, cow milk, enzyme-linked immunosorbent assay

## Abstract

**Background and Aim:**

Aflatoxins are metabolites of molds that exert potentially toxic effect on animals and humans. This study aimed to investigate the occurrence of aflatoxin M_1_ (AFM_1_) in raw cow milk collected during 1 year (2016-2017) from different regions of Algeria and risk factors associated with the contamination.

**Materials and Methods:**

During the survey period, 84 samples of raw milk were collected in three regions of Algeria (northeast, north center, and northwest) during four seasons. AFM_1_ levels were analyzed by competitive enzyme-linked immunosorbent assay.

**Results:**

AFM_1_ was detected in 39 (46.43%) samples (total mean concentration, 71.92 ng/L; range, 95.59-557.22 ng/L). However, the AFM_1_ levels exceeded the maximum tolerance limit set by the Food and Drug Administration in the USA (500 ng/L) in only 1 sample (1.19%). Statistical analysis revealed significant differences (p˂0.005) between AFM_1_ levels in milk samples collected in the spring and autumn. The mean AFM_1_ levels in samples collected in the spring were significantly higher than those in samples collected in autumn.

**Conclusion:**

The survey indicates that farmers involved in milk production should be made aware of the adverse effects of aflatoxin contamination in animal feed. A systematic control program of supplementary feedstuff for lactating cows should be introduced by the public health authorities.

## Introduction

Aflatoxins are secondary metabolites of molds, mainly produced by *Aspergillus flavus*, *Aspergillus parasiticus*, and *Aspergillus nomius* [1,2]. They contaminate a wide variety of food and agricultural products, such as cereals, seeds, grain, and silage [3]. Aflatoxins are one of the most widely studied groups of mycotoxins due to their recognized toxicity, and hepatotoxic, mutagenic, teratogenic, immunosuppressive, and neoplastic effects [4]. Although 17 aflatoxins have been isolated to date [5], only five of them are well known and studied extensively from the toxicological point of view. These are aflatoxin B_1_ (AFB_1_), B_2_, G_1_, G_2_ and M_1_. AFB_1_ is the most important and potent natural carcinogen and has been classified by the International Agency for Research on Cancer in Group 1 of human cancer-causing compounds [5,6]. The most rapidly formed metabolite of AFB_1_ is aflatoxin M_1_ (AFM_1_) produced by the liver in cattle following ingestion of the parental toxin in contaminated feed [7]. Similar to other aflatoxins, AFM_1_ has been classified in Group 1 as carcinogenic to humans since sufficient evidence exists for its hepatocarcinogenicity in humans [5]. Approximately 0.5-5% of AFB_1_ is transferred in milk as AFM_1_. After ingestion of cattle feed contaminated with AFB_1_, AFM_1_ is detectable in milk within 3 d and becomes undetectable within 4 d after the contaminated feed is withdrawn [8,9].

Milk is considered a staple food for humans of all age groups due to its high nutritional value [10]. It plays a central role in human diet and therefore holds a great economical significance on the global nutritional level [11]. The rate of AFM_1_ excretion in milk (carryover) depends on different nutritional and physiological factors, such as feeding regimen, ingestion and digestion rates, animal health, hepatic biotransformation capacity, and lactation period [12-14]. Furthermore, AFM_1_ is heat stable in raw processed milk and dairy products and is not completely destroyed by pasteurization, sterilization, and other food processing procedures [15].

Recently, several surveys concerning AFM_1_ contamination and its presence in milk and dairy products have been conducted in Croatia [16-18], Serbia [19], Italy [20-22], France [23], Spain [24], Greece [25], Iran [26-28], Pakistan [29-31], and Turkey [32,33]. Due to the potential toxicity of AFM_1_, most countries have set maximum permissible levels for AFM_1_ in milk and milk products. In the European Union (EU), the maximum legal level of AFM_1_ is 0.050 µg/kg for milk and dairy products [34]. The Food and Drug Administration (FDA) in the USA and the Codex Alimentarius set an action level for AFM_1_ in milk is 500 ng/L [35,36]. Unfortunately, the maximum permissible level of AFM_1_ in milk has not been established in Algeria. To the best of our knowledge, only one preliminary study of AFM_1_ milk contamination has been performed, and in only one district, in Algeria [37].

This study aimed to evaluate the concentration of AFM_1_ in raw cow milk collected during a 1-year period (2016-2017) in different regions in Algeria and to investigate the risk factors associated with such contamination.

## Materials and Methods

### Ethical approval and informed consent

Raw milk was collected from bulk tanks on the farms, which did not need contact with animals. The present study did not require ethics approval. Informed consent was obtained from all cattle farm owners.

### Study area

**Figure-1:**
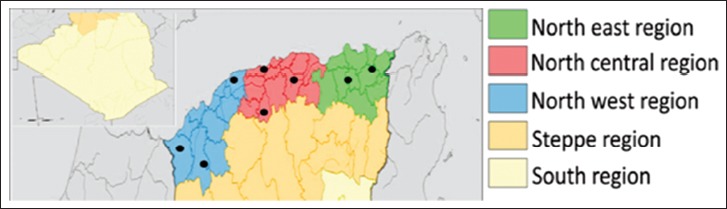
Study area map showing the sample collection regions [Source: Map prepared by the authors].

Algeria has a surface area of 2,147,570 km^2^ and is inhabited by more than 42 million people. It is positioned between the latitude 19°S and 37°N and longitude 9°W and 12°E. More than 60% of the Algerian population lives in the northern hilly areas. Algeria is divided into 48 administrative districts. For the purposes of the current study, the country was divided into five regions (Figure-1): North region (35.3°N-36.8°N and 1°E-4.7°E), with 10 districts; northwestern region (35°N-36.3°N and 2°W-1°E), with 10 districts; northeastern region (35.3°N-37°N and 4.7°E-8.5°E), with 9 districts; steppe region (33°N-35.3°N and 2°W-8.5°E), with 11 districts; and south (Sahara) region (19°N-33°N and 8.8°W-12°E), with 11 districts. The steppe and Sahara regions were excluded from the study because they are not cattle-breeding areas (sheep and goat farming predominates in these two regions).

### Sample collection and preparation

For the study, 84 samples of raw fresh cow milk were randomly collected from dairy cattle farms from August 2016 to July 2017 (n=23, northeast; n=22, center north; and n=39, northwest). Raw milk was collected from bulk tanks on the farms. The individual sample size was approximately 0.5 L. Samples were transported to the laboratory in iceboxes and stored frozen at −18°C until analysis. Personal interviews of the cattle farm owners enabled the collection of information (in the form of a questionnaire) about the number of cattle per farm, feeding system, feed storage practices, and sample collection date.

We have considered that on smallholder farms, a number of cows were ≤40, and on large farm, the number of dairy cows was ≥41.

Season-wise distribution was done as follows:

- Winter: December 2016-January 2017-February 2017
- Spring: March 2017-April 2017-May 2017
- Summer: August 2016-June 2017-July 2017
- Autumn: September 2017-October 2017- November 2017.

### Sample analysis

Milk samples were analyzed using enzyme-linked immunosorbent assay (ELISA). Before the analysis, milk samples were thawed at 4°C for 30 min. Then, 5 mL of the sample was centrifuged for 10 min at 3000× *g* at 4°C. After centrifugation, the lower (serum) layer was collected by aspiration with a Pasteur pipette. Next, 0.4 mL of milk serum was mixed with 0.1 mL of 100% methanol (4:1) and used for ELISA. AFM_1_ levels were determined by direct competitive ELISA using the AgraQuant^®^ AFM_1_ Plus ELISA (100/2000 ng/L) kit supplied by Romer Labs^®^ Singapore Pte. Ltd. (Singapore), following the manufacturer’s instructions.

All standards and samples were analyzed in duplicate. One well coated with an AFM_1_-specific antibody was used for each standard (0, 100, 200, 500, 1000, and 2000 ng/L) or sample. For the analysis, 200 µL of conjugate solution was dispensed into wells. Then, 100 µL of each standard solution or sample were placed in the appropriate dilution well and carefully mixed. The solutions (100 µL) were then placed in individual antibody-coated microwells and incubated at room temperature (18-30°C) for 20 min. Then, the liquid was poured out, and the microwell holder was tapped upside down against an absorbent paper to ensure removal of liquid from the wells. The liquid was decanted and wells were washed 5 times with a diluted wash buffer. Then, 100 µL of the substrate were pipetted into each well and incubated for 10 min in the dark. At the end of incubation, 100 µL of stop solution was dispensed into the antibody-coated wells. Sample absorbance was measured at 450 nm using a microwell plate reader (Biotek Elx800, Winooski, Vermont, USA).

### Method validation

According to the AgraQuant^®^ AFM_1_ Plus ELISA kit, the limit of detection (LOD) of AFM_1_ in fresh milk is 89 ng/L. The LOD of the method satisfied the maximum tolerance limit set by the FDA in the US (500 ng/L) [38]. Samples were considered to be positive for AFM_1_ if the levels exceeded the LOD of the assay.

To determine the efficiency of the assay, a standard solution of AFM_1_ was purchased from Sigma-Aldrich (St. Louis, MO, USA). Validation of ELISA was performed by determining the recovery and mean variation coefficient in raw milk spiked with different concentrations of AFM_1_ (100, 250 and 500 ng/L) and analyzing AFM_1_ in raw milk. The recovery of AFM_1_ in spiked milk samples was 95.6% (coefficient of variation: CV=1.23), 94% (CV=1.11), and 99% (CV=1.06) for spiked concentrations of 100, 250 and 500 ng/L AFM_1_, respectively. All experiments were repeated 5 times. The recovery rates satisfied the guidelines for recoveries set by the Codex Alimentarius Standard [39]. The standard calibration showed excellent linearity, with R^2^ value of 0.999.

### Statistical analysis

Statistical analysis was performed using SPSS software 20.0 (IBM Corp., NY, USA), at 95% significance level. The positivity rates in samples were compared using Fisher’s exact test or Chi-square test. The mean AFM_1_ levels were compared using Student’s t-test or ANOVA.

For the risk factor analysis, a univariable analysis for variable selection was first performed at p≤0.2, using the χ^2^ test or Fisher’s exact test. The variables that passed this cutoff were then analyzed by logistic regression [40]. The variables were ruled as risk factors when the odds ratio was >1 and p≤0.05.

## Results

The survey revealed high AFM_1_ levels in the raw cow milk collected in Algeria (overall mean of 71.92±28.48 ng/L). The positivity rate of AFM_1_ contamination was 46.42%. Further, from 84 (100%) tested raw milk samples, AFM_1_ levels were below LOD (89 ng/L) in 45 (53.6 %) samples; between 89 and 300 ng/L in 35 (41.7%) samples; between 301 and 500 ng/L in 3 (3.6%) samples and over 500 ng/L in 1 (1.19%) sample (Tables-1-3).

**Table-1:** Distribution of AFM_1_ levels according to the region in Algeria.

Region	Districts	Number of samples	Distribution of AFM_1_ level (ng/L)			

			Mean±SE (range)			

			<LOD*	89-300	301-500	>500
Northeast	Constantine, Mila	23	16	7	0	0
			-	112.42±19	-	-
			-	(96.87-147.83)	-	-
North center	Médéa, Tipaza, Djelfa	22	5	15	2	0
			-	154.94±45.15	453.49±6.66	-
			-	(95.59-231.17)	(448.78-458.20)	-
Northwest	Chlef, Tlemcen, Mascara	39	24	13	1	1
			-	125.35±21.28	344.99	557.22
			-	(100.58-178.48)	-	-
Overall		84	45	35	3	1
%		100	53.57	41.66	3.57	1.19

* LOD=Limit of detection, AFM_1_=Aflatoxin M_1_, SE=Standard error

**Table-2:** Distribution of AFM_1_ levels according to the farm size in Algeria.

Farm size	Number of samples	Distribution of AFM_1_ levels (ng/L)			

		Mean±SE (range)			

		<LOD*	89-300	301-500	>500
Small	47	25	19	2	1
			147.03±43.39	453.49±6.66	557.22
			(95.59-231.17)	(448.78-458.20)	-
Large	37	20	16	1	0
			118.09±23.16	344.99	-
			(96.87-183.91)	-	-
Overall	84	45	35	3	1
%	100	53.57	41.66	3.57	1.19

* LOD=Limit of detection, AFM_1_=Aflatoxin M_1_, SE=Standard error

**Table-3:** Distribution of AFM_1_ levels according to the season in Algeria.

Season	Number of samples	Distribution of AFM_1_ levels (ng/L)			

		Mean±SE (range)			

		<LOD*	89-300	301-500	>500
Winter	18	10	7	1	0
			112.40±9.65	344.69	-
			(103.91-178.48)	-	-
Spring	7	3	2	2	0
			119.08±5.31	453.49±6.66	-
			(112.25-125.67)	(448.78-458.20)	-
Summer	39	22	17	0	0
			139.15±23.87	-	-
			(96.87-231.17)	-	-
Autumn	20	10	9	0	1
			128.17±18.57	-	557.22
			(95.59-229.20)	-	-
Overall	84	45	35	3	1
%	100	53.57	41.66	3.57	1.19

* LOD=Limit of detection, AFM_1_=Aflatoxin M_1_, SE=Standard error

Based on the region of origin, the mean AFM_1_ levels in raw milk samples (ng/L) were 32.94±11.87, 152.46±44.14, and 57.05±21.67, in the northeast, center north, and northwest, respectively (Table-4). Statistical analysis revealed a significant difference in the contamination levels of milk between regions (p=0.013) (Table-4). The mean concentration of AFM_1_ was significantly higher in the center north (152.46±44.14 ng/L) than in other regions.

**Table-4:** Univariable analysis of risk factors associated with AFM_1_ positivity in cattle farm milk in Algeria.

Variable	Category	Number of samples	Number of positive samples (%)	p (c^2^ test)	Total samples (ng/L)(mean±SD)	p (t/K-W test)
Region	Northeast	23	7 (30.43)	0.017	32.94±11.87	0.013*
	Center north	22	17 (77.27)		152.46±44.14	
	Northwest	39	15 (38.64)		57.05±21.67	
Farm size	Small	47	22 (46.80)	0.473	90.16±43.02	0.032*
	Large	37	17 (45.94)		58.59±27.44	
Season	Winter	18	8 (44.44)	0.381	60.28±27.38	0.025*
	Spring	7	4 (57.14)		106.92±41.92	
	Summer	39	17 (43.59)		59.77±19.65	
	Autumn	20	10 (50)		88.79±25.34	
Overall (%)		84 (100)	39 (46.42) Positive samples (ng/L) (mean±SD)	Total mean (ng/L)	71.92±28.48	
				156.71±43.15		

* Significant difference between means (p<0.005). SD=Standard deviation, AFM_1_=Aflatoxin M_1_

Analysis of season wide distribution indicated a significant difference in the mean concentration of AFM_1_ between seasons (p=0.025). The mean AFM_1_ levels in samples collected in the spring (106.92±41.92 ng/L) were significantly higher than those in samples collected in the autumn (88.79±25.34 ng/L), summer (59.77±19.65 ng/L), or winter (60.28±27.38 ng/L) (Table-4).

Considering the farm size, AFM_1_ levels were significantly higher on smallholder farms (90.16±43.02 ng/L) than on large farms (58.59±27.44 ng/L) (p=0.032) (Table-4).

## Discussion

Mycotoxins pose a serious health threat to humans and animals. In the current study, we aimed to evaluate the distribution of AFM_1_ contamination levels in raw milk across Algerian and to investigate the risk factors associated with such contamination.

To the best of our knowledge, only one study on milk contamination by AFM_1_ in Algeria has been published, concerning the city of Constantine (in the northeast of the country) [37]. In the study, AFM_1_ was detected in 5 (11%) out of 47 samples, at levels ranging from 9 to 103 ng/L, with one sample exceeding the limit of 0.050 µg/kg set by the EU. In the current study, we observed 46.42% positivity rate of AFM_1_ contamination (toxin levels exceeding 0.050 µg/kg EU limit) (Table-1), with the total positive mean of 71.92±28.48 ng/L, considering only the positive samples (containing 95.59-557.22 ng/L) (Table-1). The high occurrence of AFM_1_ in the investigated cow milk samples may be associated with the notion that cows are kept in local dairy farms and fed compound rations or silage stored under inadequate conditions. This can lead to areas highly contaminated with toxigenic *Aspergillus* fungi and a consequent aflatoxin formation [41]. However, the incidence of AFM_1_ contamination reported in the current study was lower than the incidence in the neighboring Morocco, where AFM_1_ contamination of 48 (88.8%) out of 54 pasteurized milk samples and ranging from 0.001 to 117 ng/L (mean value of 18 ng/L) was reported [42].

In the present study, AFM_1_ levels in 38 out of 39 samples were below the maximum action limit established by the FDA and Codex Alimentarius (500 ng/L); the limit was exceeded in only 1 sample (1.19%). Further, the detected AFM_1_ contamination in milk samples collected in Algeria in the current study was relatively lower than that in milk produced in other countries. Tomašević *et al*. [19] analyzed 678 raw milk samples in Serbia during the years 2013-2014 and showed that AFM_1_ levels in 56.3% and 24.6% samples exceeded the maximum EU and USA set limits, respectively, with the mean AFM_1_ levels of 282 ng/L. More recently, in a study from Pakistan, AFM_1_ was detected in 143 (91.7%) out of 156 fresh milk samples analyzed, with the mean level of 342.2 ng/L, and with 125 (80.1%) and 51 (32.7%) samples containing more AFM_1_ than the maximum EU and USA set limits, respectively [29]. Collectively, these observations indicate that AFM_1_ contamination levels in milk vary among countries. These variations could be associated with different methods for toxin detection and differences in the forage and feed quality, cow diet, geographical location, climate and seasonal variations, genetic variation in dairy cows, farming systems, and feed storage [31,43,44].

The current study revealed considerable variations in AFM_1_ contamination rate in raw milk samples from different regions in Algeria. The detected sample positivity was 30.43% in the northeast, 77.27% in the center north, and 38.64% in the northwest. These variations may be linked to geographic and climatic differences [45]. It has been reported that the high temperature associated with climate change supports mycotoxin contamination [46].

Further, in the current study, the highest AFM_1_ mean levels were recorded in the spring (106.92±41.92 ng/L) and autumn (88.79±25.34 ng/L) (Table-4) that could be explained by very hot summer, severe drought, warm autumn, and a lack of rain during the winter season recorded in most parts of Algeria in the years 2016-2017 [47,48]. Severe drought may increase the risk of aflatoxin contamination [49]. Indeed, according to the studies from Croatia, 33% of cow milk samples collected in the eastern region during spring [18] and 9.32% samples in autumn [17] exceeded AFM_1_ levels established by the EU. We here showed that, in the autumn, AFM_1_ levels in only 1 milk sample (1.19%) exceeded the Codex Alimentarius and USA set maximum (500 ng/L).

Finally, the survey conducted in the current study revealed that AFM_1_ levels in milk samples from small farms were higher than those from industrial farms (Table-2). That was consistent with the observations in the field and could be explained by the notion that good storage practices and hygiene standards are not properly observed on traditional farms. In addition, farmers are not aware of the risk of contamination of animal feed by mycotoxins. Ideally, the study should be repeated in the regions in later years as well, and more farms should be sampled and on different periods.

## Conclusion

The incidence of AFM_1_ in milk is a serious public health concern in Algeria, especially for children who are more susceptible to the effects of AFM_1_ than adults. This creates a major health risk to the Algerian population. The levels of contamination found in samples tested in the current study exceeded the maximum tolerable levels set by the EU and the USA. However, the high AFM_1_ levels were probably a consequence of the usage of AFB_1_ contaminated feed of dairy cows. The most effective way of controlling AFM_1_ in milk is monitoring AFB_1_ presence in the feed. The potential health risks of AFM_1_ may be reduced by increasing farmer awareness, improving feed storage practices, and intensive self-controls in the dairy industry. Further studies should be conducted to obtain more data regarding AFM_1_ contamination of milk in Algeria. It is also important that the competent authorities establish the maximum permissible levels of AFM_1_ in milk and milk products in Algeria.

## Authors’ Contributions

SM and MD designed this study and analysis in the laboratory. SM and MD collected samples. SM, MD, CB, MK, and MHB drafted, revised the manuscript, analyzed the data, and approved the final manuscript. All authors read and approved the final manuscript.
